# BASH: badmouthing, attitudes and stigmatisation in healthcare as experienced by medical students

**DOI:** 10.1192/pb.bp.115.053140

**Published:** 2016-04

**Authors:** Ali Ajaz, Rhodri David, Damien Brown, Melanie Smuk, Ania Korszun

**Affiliations:** 1 East London NHS Foundation Trust; 2 Barts and The London School of Medicine and Dentistry

## Abstract

**Aims and method** We used an online questionnaire to investigate medical students' perceptions of the apparent hierarchy between specialties, whether they have witnessed disparaging comments (‘badmouthing’ or ‘bashing’) against other specialists and whether this has had an effect on their career choice.

**Results** In total, 960 students from 13 medical schools completed the questionnaire; they ranked medical specialties according to the level of badmouthing and answered questions on their experience of specialty bashing. Psychiatry and general practice attracted the greatest number of negative comments, which were made by academic staff, doctors and students. Twenty-seven per cent of students had changed their career choice as a direct result of bashing and a further 25.5% stated they were more likely to change their specialty choice. Although 80.5% of students condemned badmouthing as unprofessional, 71.5% believed that it is a routine part of practising medicine.

**Clinical implications** Bashing of psychiatry represents another form of stigmatisation that needs to be challenged in medical schools. It not only has an impact on recruitment into the specialty, but also has the wider effect of stigmatising people with mental health disorders.

Choosing a career specialty is one of the most important decisions that medical students will make and one that will shape the rest of their working lives. Some students decide early on, or even come into medical school with a good idea of what career they would like to pursue,^1^ yet for most students the specialty choice is made during medical school,^2^ with some remaining unsure into their final year and even as foundation year doctors.^3^ This choice is influenced by many factors, in particular the teachers the students encounter,^4^ experience during clinical placements,^5^ desired work-life balance,^6^ and also by gender^3^ and personality.^7^

Psychiatry continues to face a worldwide problem with recruitment. In the UK, the Royal College of Psychiatrists has maintained an active recruitment programme for several years, but rates of students interested in psychiatry as a career remain low at 4-5%,^8^ which is insufficient to meet future needs. A variety of approaches have been used to improve recruitment. Time and effort has gone into promoting psychiatry, and the misperceptions that put students off, such as that people ‘don't get better’^9^ or psychiatry ‘isn't proper medicine’,^10^ have been successfully challenged. As psychiatrists, we constantly strive to improve our profile, our teaching and our support and mentorship of students who are interested in psychiatry. But even though psychiatrists become involved in setting the medical curriculum and integrating psychiatry into mainstream medical teaching, it is not so easy to control what else is going on in the, now long-recognised, ‘hidden’ curriculum.^11^

One aspect of this hidden curriculum is the apparent hierarchy between different specialties, with specialists making disparaging comments and ‘badmouthing’ or ‘bashing’ each other's specialty.^12,13^ A stable prestige hierarchy exists among medical specialties, with certain ones, such as surgery and cardiology, consistently ranked at the top and others, such as dermatology and psychiatry, consistently resting at the bottom.^14,15^ This has led to many enduring stereotypes of ‘macho surgeons’, ‘fluffy psychiatrists’, ‘lazy dermatologists’ and ‘useless general practitioners’.^12,16^ Should these be regarded as just some fun and perhaps part of the medical tradition to which succeeding generations of medical students are inducted? Or do these attitudes actually have a detrimental effect on the students' perception and choice of some specialties?

In one large US study,^17^ 76% of students had heard badmouthing of their career choice specialty and 17% stated that this had made them alter their career choices. In a more recent survey of third-year US students,^18^ family medicine was the most ‘bashed’ specialty, but psychiatry was not far behind, with 39% of students stating that they had heard disparaging comments about psychiatry. There are no data available on how common this practice is in UK medical schools and whether it affects our students' career choices. We thus undertook an online questionnaire study to measure: (a) how frequently British medical students encounter badmouthing of different specialties and (b) whether witnessing badmouthing affects their choice of career specialty. Ethics approval was obtained from the Barts and The London School of Medicine and Dentistry and the Research Ethics Committee at Queen Mary University of London (Ref: QMREC 0987).

## Method

We conducted a nationwide questionnaire survey of students at UK medical schools. An invitation letter and weblink to the online survey was sent to the relevant administrators at all 32 UK medical schools asking for it to be distributed to all their students (years 1-5 and students intercalating degrees), with a reminder email sent 2 weeks later. The survey remained open for 6 months and participation was entirely on a voluntary basis. Students were informed in the invitation letter that the responses would be anonymous and all data were also stored anonymously as stipulated in the ethics approval.

### The questionnaire

The online questionnaire was made available through the Survey Monkey website (www.surveymonkey.com) and questions were based on previous studies.^18^ In the instructions for completing the questionnaire badmouthing and bashing were defined as: ‘distinctly negative feedback and comments directed by members of one medical specialty toward other specialties’. Participants were asked to complete demographic items (age, gender, medical school and year of study). Students were then asked: (a) to rank a list of medical specialties according to the level of badmouthing or negative comments that they attract (most negative comments to fewest negative comments); (b) how often the students themselves had made negative comments about these specialties (very frequently to never); and (c) to indicate the frequency of negative comments about other specialties that had been made by specific groups of individuals (academic staff, non-academic staff, doctors during clinical placement and other medical students). Participants were encouraged to provide examples of such statements in the free-text boxes. Participants were also asked to rank their level of agreement with ten statements relating to future career choice (Box 1).

Although this was not a qualitative study, participants also had the opportunity to make any additional comments on the topic of specialty badmouthing in a free-text box. These responses were reviewed to explore whether any specific patterns emerged. These were categorised and combined into overarching themes to provide further understanding of the quantitative data.

The data contained missing observations. We explored the missing data patterns and looked at predictors for being missing using logistic regression models. No strong patterns or predictors were found, which suggested that we could use complete case analysis without introducing bias. Analysis was undertaken using Fisher's exact test, Friedman test and Spearman's rank correlation to handle the ordinal nature of the data.

**Box 1** Questionnaire statements relating to students' career choice

1. I've changed my career option due to hearing negative comments being made about my specialty of choice.
2. When asked about my specialty choice, I've avoided the question or misled others to avoid being treated differently.
3. I'd be more likely to change my specialty choice if I witnessed it being badmouthed.
4. Badmouthing wouldn't affect my specialty choice.
5. I think badmouthing other specialties is unprofessional.
6. Badmouthing other specialties is just a bit of fun and is part of working as a doctor.
7. I think badmouthing would affect whether somebody chooses a certain specialty.
8. Badmouthing other specialties is always going to be a part of practising medicine.
9. Doctors that badmouth other specialties are insecure about their own career choice.
10. There's an unspoken hierarchy of specialties in medicine.

## Results

A total of 960 medical students completed the survey. Although every medical school in the UK was invited to take part, there was no mechanism for checking whether the administrator had forwarded the survey link to students and replies were received from students attending 13 universities. The distribution of students throughout the undergraduate medical course is shown in Table 1.

**Table 1:** Respondent demographics (*n* = 960)

	*n* (%)
Gender	
Male	346 (36.0)
Female	614 (64.0)

Age, years	
18–20	248 (25.8)
21–29	673 (70.1)
30–39	34 (3.5)
Over 40	5 (0.5)

Year of study	
1	146 (15.2)
2	131 (13.6)
3	145 (15.1)
4	254 (26.5)
5	227 (23.6)
Intercalated BSc	23 (2.4)
Other	34 (3.5)

The survey did not force students to rank specialties as described in the accompanying instructions to this item. Thus, not all students followed the instructions, and the sample size for analysis for this item was decreased to 483 for students who had ranked correctly. Demographics were similar for correctly and incorrectly ranking students. The other analyses kept the cohort at *n* = 960.

From the list of eight medical specialties provided, students reported that both psychiatry and general practice attracted the greatest number of negative comments. The difference in ranking over all groups was statistically significant (Friedman test *P*<0.001) and the mean ranking for each was as follows (1 attracts the most negative comments, 8 attracts the fewest negative comments): general practice 2.20, psychiatry 2.29, radiology 4.04, surgery 4.55, anaesthetics 5.08, obstetrics and gynaecology 5.13, emergency medicine 6.35 and hospital medicine 6.37. Psychiatry was significantly different (Wilcoxon signed rank test *P*<0.001) from all specialties except general practice.

Participants reported hearing negative comments about different specialties made by other medical students, academic staff members (both clinical and non-clinical) whom they had encountered at university, and doctors of all grades during their clinical placements (Table 2). The highest frequency of negative comments was attributed to other medical students.

**Table 2:** Negative comments about specialties (*n* = 960)

	Frequently, %	Sometimes, %	Never, %
*How often do you hear people make negative comments about other specialties?*			
Medical students	44.7	52.1	3.2
Consultants during placements	32.8	57.1	10.2
Registrars during placements	26.8	63.1	10.2
Junior doctors during placements	20.1	70.0	9.9
Academic staff			
Medical (e.g. professors, clinical lecturers)	19.6	72.8	7.6
Non-medical (e.g. non-clinical lecturers)	10.5	75.4	14.1

*How often do you personally make negative comments about the following specialties?*			
Surgery (e.g. ENT, orthopaedics)	9.6	56.4	34.1
General practice	9.1	59.3	31.6
Psychiatry	7.3	57.0	35.7
Radiology	3.8	50.6	45.5
Obstetrics and gynaecology	3.2	46.8	50.1
Anaesthetics	2.8	44.5	52.7
Hospital medicine (e.g. cardiology, respiratory medicine)	1.5	43.2	55.3
Emergency medicine	1.1	35.6	63.4

ENT, ear, nose and throat.

Students were asked how frequently they themselves had made comments against other specialties. Psychiatry, general practice and surgery attracted the most critical comments from the student respondents. Examples of disparaging comments were: ‘psychiatrists are not actual doctors’, ‘psychiatry – not real medicine’, ‘psychiatrists are crazy themselves’, ‘you don't need a medical degree to be a GP’, ‘people become GPs when they fail all other exams’.

The association between the frequency of students hearing negative comments and the frequency of students making negative comments was explored with a Spearman's rank correlation. Students significantly (*P*<0.001) reported higher frequencies of making more negative comments with higher frequencies of hearing negative comments.

Participant gender was not associated with making or hearing negative comments about specialties. However, there was a significant association (Fisher's exact test) between age and negative perceptions of psychiatry (*P* = 0.03), surgery (*P* = 0.037) and anaesthetics (*P* = 0.004), namely negative perceptions decreased with increasing age.

There was a significant association (Fisher's exact test) between year of study at medical school and ranking badmouthing for psychiatry (*P* = 0.001), general practice (*P* = 0.02), surgery (*P* = 0.008) and anaesthetics (*P* = 0.001). For psychiatry, negative perceptions were least frequent in the first year of medical school and most frequent in the fifth year, whereas for anaesthetics the opposite was true.

Although 80.5% of students condemned badmouthing as being unprofessional, 71.5% believed that it is an ever-present part of practising medicine; 57.3% viewed it as ‘just a bit of fun’; 27.1% agreed with the statement that doctors who ‘badmouth’ other specialties ‘are insecure in their own career choice’; and 74.0% agreed that there is an unspoken hierarchy of specialties in medicine.

When exploring the influence of badmouthing on career choice, 27.0% of students agreed that they had changed their career choice as a direct result of negative comments made about them and a further 25.5% said that they were more likely to change their specialty choice if they had witnessed it being ‘badmouthed’. Over a third (36.8%) had feared being treated differently by others when asked about their specialty choice and therefore resorted to either avoiding the question or providing misleading answers. Two-thirds (66.2%) agreed that badmouthing against a particular specialty would affect a student's decision to choose it as a career.

### Thematic review of comments

The final question of the survey asked students to make any additional statements related to specialty badmouthing. A total of 146 students made comments and these were reviewed by two researchers (A.A. and A.K.) and grouped into four main categories according to identified themes.

#### 1. Acceptance of badmouthing

The majority of comments (40.4%), either directly or indirectly, minimised any negative connotations associated with badmouthing other specialties or the impact on students' future career choices. The most frequent comments stated that badmouthing was nothing but harmless fun and was done without any maleficent intentions. There was a real sense among some students that badmouthing was beneficial as a source of conversation and bonding within clinical teams. There was some recognition that some medical students might be influenced by the negative comments about certain specialties, but this was dismissed as being due to their own insecurities about their career choice rather than being affected by the remarks.

‘Almost every specialty badmouths at least one other – in a way this is good as it allows us as medical students to make an informed choice.’

‘Playful banter between different specialties … can be a topic of conversation which bonds medical staff together.’

‘I feel that if you like a specialty enough, what people say is never going to change how you feel.’

‘Harmless bit of fun … more like teasing than anything, although it could offend someone who is insecure about their career choice.’

#### 2. Negative effects of badmouthing

Some students admitted to having rejected certain specialties as career options because of experiencing negative comments, either about the nature of the work or personal qualities of doctors who choose that field. The most commonly mentioned specialties were psychiatry, general practice and dermatology. Other students reported that they purposefully kept their career choice to themselves because of fear of discrimination by their peers or clinicians at their clinical placements. Some participants condemned all types of badmouthing and believed it to be unprofessional.

‘Badmouthing of GPs has put me off going into it as much as it seems less respected professionally and doctors don't give as much credit to [the GPs'] findings as they should.’

‘I have heard consultants refer to GPs like idiots … It makes it a really difficult career to feel good about going into and is making me wonder whether after all the hard work at medical school I want to go into a career where I'm not respected and I'm almost a level below hospital doctors.’

‘If I am asked by a clinical member of staff … I very often do not tell them that I have an interest in psychiatry, as I feel that this may mean that I am judged automatically about my knowledge, skills and attitude towards medicine in general.’

‘It's unethical and therefore fundamentally undermines a health professional's code of conduct.’

#### 3. Grey area of acceptability

Some students raised the issue of distinguishing degrees of badmouthing that are acceptable. Some believed that there was a ‘fine line’ between comments made in jest and those that were damaging to other specialties. There were some views that badmouthing at undergraduate level was nothing but ‘harmless banter’, whereas similar comments by qualified doctors were considered to hold more weight and be more influential and therefore unprofessional. Others stated that any badmouthing was both harmless and acceptable in medical circles, however, anything that extended to (or was overheard by) members of the general public was inappropriate. One student stated that medical students were relatively immune to the negative effects of badmouthing because they were still ‘explorers’ (on the path of choosing a career specialty) and that doctors in training were the ones who were more vulnerable to changing their choice of career in response to badmouthing by their more senior colleagues.

‘Most of the badmouthing heard within the student generation is usually a bit of fun and not meant in seriousness. The genuine badmouthing is heard at higher levels, i.e. senior registrar or consultant.’

‘I think it is important to distinguish between badmouthing which can be unprofessional and banter between specialties which is part of the job – there is a fine line.’

#### 4. Addressing bashing

There were comments that specialty badmouthing is an ingrained and accepted part of the culture in medicine for students and doctors. Thus, there was an element of helplessness expressed by some students in considering how to overcome this practice. The main suggestions focused on raising awareness of its negative effects and the need to educate students and doctors of the value of each specialty and how any form of badmouthing (regardless of the intention) is a breach of medical professionalism.

‘ … badmouthing is so much part of what doctors and surgeons talk about that it would be difficult to stop it completely, but maybe raising awareness that it does affect which specialty students may go into in the future would make them think about what they say and how they say it more.’

‘I don't see a way it can be altered. It's tradition – hard to change.’

‘There ought to be efforts made to challenge the mentality of the “hierarchy of specialties” and broaden the horizons of what medical students think about different specialties.’

## Discussion

The sample of students taking part in this study came from 13 medical schools and included students from all medical school years, with a representative age range. The larger proportion of women in the sample makes it less representative of the student population but is consistent with generally greater participation of women in online surveys.^19^

In this sample of UK medical students, the specialties significantly attracting the most negative comments were psychiatry and general practice, similar to the findings in a group of third-year US students.^18^ The more senior the students, the more likely they were to have encountered psychiatry bashing and fifth-year medical students reported the highest levels. This may just reflect the fact that they have been around longer so have had more opportunities to come across this, but it could also indicate that this behaviour becomes more ingrained as students progress through medical school. There was no gender difference found in this sample.

Making disparaging comments was not limited to any particular group and students reported university academic staff (both clinical and non-clinical) and doctors of all grades during their clinical placements bashing their colleagues from other specialties. However, it was other medical students that were reported to make the most comments. This could be just because students encounter many more students than they do senior doctors and academics, but, nevertheless, this supports the fact that bashing is alive and well among our next generation of doctors. Survey participants also admitted that they themselves had made negative comments about certain specialties and there was a significant association between making and hearing badmouthing. Although career choice is obviously an important topic for discussion, it would be disappointing if this consists only of a bashing competition.

Psychiatry and general practice were the most bashed specialties, but other specialties also attracted an array of disparaging comments that reflect the stereotyping that still exists within medicine, such as ‘surgery – workaholic and rubbish with patients’, ‘radiology – boring’, ‘surgeons lacking social skills, being obsessed with cutting, thinking they were God's gift’, ‘dermatology – derma-holiday’. Clearly, students show some confusion about this behaviour: 81% condemned it as unprofessional, but 72% believed that it is an integral part of medicine and over half thought that it is ‘just a bit of fun’. In the free-text remarks, students talked about the need to take part in bashing to ‘bond within clinical teams’, suggesting that part of the hidden curriculum involves social coercion to ‘bash’ others. There are further justifications of this behaviour in that it only affects those who are ‘insecure about their choice of specialty’ – the latter of course applies to anyone who is still in the process of choosing.

But what is a bit of fun to some students clearly has a major impact on others (27%) who reported that they had changed their career choice as a direct result of negative comments. Over a third of students avoided disclosing their choice as they feared being treated differently and two-thirds agreed that bashing could affect students' decisions about careers. It is clearly unacceptable for students not to feel able to be open about their career choice in medical school. But students also highlighted the grey area between degrees of badmouthing with a ‘fine line’ existing between comments that are made as ‘harmless banter’ and those that could be damaging to other specialties. An important question is where should that line be drawn? Also, are there certain specialties that are more prone to bashing, preventing students from disclosing their interest in them because of attached stigma or perceived low status? It was clear that disparaging comments made by qualified doctors held more weight than those made by students.

It is well known that there is stigma associated with psychiatry and psychiatrists.^20,21^ But the disparaging comments made about the specialty (e.g. ‘not real medicine’) are not just stigmatising to psychiatrists, but have the wider effect of stigmatising people with mental health disorders (e.g. ‘psychiatry – worse than their patients’, ‘psychiatrists are crazy themselves’). Both psychiatry and general practice are currently experiencing a crisis of low recruitment. Mental health disorders are highly prevalent in our population and this will have a profound effect on those affected. If mental health services are unavailable because there is a shortage of psychiatrists, then further burden will fall on general practice, which is in turn suffering a recruitment crisis and widespread bashing. At a time when public support is growing for challenging the stigma associated with psychiatric conditions and the government has stated its commitment to achieving parity of services for people with mental health disorders, it is disheartening that we still have to overcome the stereotyping and stigmatisation of psychiatry by our colleagues from other medical specialties.

### Study limitations

There could have been bias in the students who responded. Because ethics allowed us only to send the link to the named administrator at each medical school office, we could not make sure that the link was forwarded to all available students. We are therefore unable to say accurately what the response rate was. However, the spread in age and year of study was good and 64% women in the sample, although also a limitation, is typical of such online surveys. Furthermore, there is a possibility that students could have responded to the questionnaire more than once, although there was no incentive for them to do so.

The questionnaire contained a short explanation of what is meant by bashing but different students may interpret this term differently.

Only some of the respondents gave free-text responses and other than identifying the four thematic groupings there was no formal qualitative analysis of these responses. However, the identification of the themes is useful in interpreting the quantitative findings and to inform future studies.

### Conclusions and future work

Despite the limitations of this initial study examining the prevalence and effects of specialty bashing on career choice, there is clear evidence of bashing throughout the medical school course by all groups of students and teachers. Although some students see it as an integral part of medical school life, a third are put off their career choices. More work needs to be done to look in detail at the effects of bashing and to confirm whether these findings are generalisable. But there is clearly no time to lose in challenging bashing, which represents another form of stigmatisation of psychiatry and those with psychiatric disorders.

We need to find an effective means of challenging the belief that this behaviour is an ingrained and accepted part of our medical culture. The fact that it is mainly students who are engaging in it presents an immediate opportunity for us to have a grassroots campaign that addresses these questions and challenges the perception that it is fine to disparage certain specialties. This can only be achieved through open discussion and by raising awareness of the detrimental effects of bashing. Where should the line be drawn between friendly banter and abuse? How does bashing fit in with medical professionalism that requires showing respect for all patients and for all colleagues in our own and other specialties? Most importantly, how should students challenge bashing when they come across it? Students need to feel empowered to challenge this because there is clear and open condemnation of this behaviour by their medical school.
